# School Nursing Data Collection During COVID-19

**DOI:** 10.1177/1942602X20960214

**Published:** 2020-11

**Authors:** Erin D. Maughan, Martha Dewey Bergren

**Affiliations:** NASN Director, Silver Spring, MD; College of Nursing University of Illinois, Chicago, IL

**Keywords:** school nurse data, NASN framework quality improvement, COVID-19, school nurse value, *Every Student Counts!*

## Abstract

COVID-19 has affected the 2020-2021 school year for everyone and thrust school nurses into the spotlight. Some school nurses are too overwhelmed to even think about data; others want to collect data differently to illustrate the value of the role of the school nurse. This article provides guidance on data collection during this unique time period. The article is based on a blog originally posted on National Association of School Nurses’s website.

Coronavirus disease 2019 (COVID-19) has put the health of students front and center and has thrust school nurses into the spotlight. School nurses are challenged and asked to accept responsibilities on a scale never before expected (McDonald, 2020). As schools scramble to put physical distancing in place, and other learning going virtual, many school nurses ask if they should bother collecting data. Other school nurses realize the need to collect different data to reflect the escalation in the level of care. These school nurses see an opportunity to put the valuable role of school nurses into a brighter spotlight. This article provides insight into practical data points that school nurses can collect to reflect their contributions during COVID-19.

Documentation and data collection are fundamental practices of school nurses (American Nurses Association [ANA] & National Association of School Nurses [NASN], 2017) and are practice components in NASN’s *Framework for 21st Century School Nursing Practice* in the principle of quality improvement (NASN, 2020). Documentation provides proof of school nurses contributions to student health (Nicholson & Johnson, 2020). Data collection reveals trends and is the basis of evidence-based school nursing practice (Lepkowski, 2018). Pre-COVID-19, researchers found that a school nurse can save principals 13 hours per week (Baisch et al, 2011); and school nurses can spend over an hour on one call with concerned parents (Bergren, 2016). How much more time would a school nurse save principals and teachers now? Data would tell us; data from frontline school nurses.

## Start Small

Never let a crisis go to waste! It is often when things are turned upside down that innovation and new habits are generated. If you have not collected data before, this is the year to start. Begin with one key activity or data point (Hinkle & Maughan, 2020). What does your principal ask you the most about? Or what vital skill does your principal not understand about school nurses? Prioritize skills that reflect nursing expertise and critical thinking (ANA & NASN, 2017). Make that your 2020-2021 data point. Another option is to begin collecting data points included in NASN’s (n.d.) *National School Health Data Set: Every Student Counts!* Currently there are data points related to workforce, number of students with chronic conditions, and health office visits.

## COVID-19

If you already collect data, you may want to think about the novel activities you will be doing this year (especially if they are different than in past years). Remember to focus on data points that illustrate critical thinking and skills only a school nurse can do. Examples may include the following:

- Number of virtual visits
- Number of COVID-19 educational sessions broken down by audience: students, staff, and parents
- Number of parent communications (phone, email, virtual meetings)

○ Time on calls and in parent meetings

- Number of COVID-19 contacts traced
- Number of students identified by the school nurse for being at risk

○ Interventions delivered by the school nurse to address need, including population mental health

- Percentage of students contacted by school nurse (broken down by race/ethnicity, free/reduced lunch, disability)

○ And specific outcomes, such as improved engagement or attendance, improved health, or other needs met

- Number of unengaged or chronically absents students, and the percentage of those students contacted by school nurse

○ And specific outcomes from your interactions such as resources provided or referrals made

- Number of COVID-19 screenings (track the types of symptoms exhibited to warrant the screening)

○ Number of COVID-19 cases

- Number of times an isolation room was set up for a suspected case—number of people in the isolation room simultaneously
- Number of teacher communications (phone, email, virtual meetings)

○ Time on calls and in teacher meetings

- Number of administrator communications (phone, email, virtual meetings)

○ Time on calls and in administrator meetings

- Number of health department communications (phone, email, virtual meetings)

○ Time on calls and in health department meetings

- Number of healthcae provider communications (phone, email, virtual meetings)

○ Time on calls and in provider meetings

- Number of school nurse communications, internal and external for consultation (phone, email, virtual meetings)

Whatever data point you decide to collect, set up a system to streamline the data collection process so that accurate data is collected (Hinkle & Maughan, 2020). This could be on a tracking form you create on paper, or ideally using an electronic health record (Lepkowski, 2018). Document in real time and keep accurate records. Guesstimates or inaccurate data are not helpful and actually hurts school nurses’ credibility (Bergren, 2016; Guthrie, 2019).

Be sure to share your data at the end of the school year (Wysocki & Maughan, 2019). When presenting your data, provide context so that educators understand the coordination and nursing skills used in these activities. In other words, be sure they understand the school nurse’s contribution so that they cannot say, “well, a secretary could make phone calls.” What process or outcomes are different because the school nurses made the calls?

This new school year brings a lot of unknowns that increases stress. During crises we often get overwhelmed because our glass is overflowing already. COVID is that crisis. Yet it is during a crisis that we are forced to identify the most critical activities we must perform. We create a mind shift that expands the understanding of our own potential. With so much shifting to a “new normal” let us make 2020-2021 the Year of the School Nurse (to coincide with 2020 being the Year of the Nurse). Let us make 2020-2021 the year school nurses shift to the new normal and put data front and center.■
